# Interaction of type 2 diabetes mellitus with Chromosome 9p21 rs10757274 polymorphism on the risk of myocardial infarction: a case–control study in Chinese population

**DOI:** 10.1186/1471-2261-14-170

**Published:** 2014-11-27

**Authors:** Liu-wei Zhang, Jian-ping Li, Fang-fang Duan, Zhi-ke Liu, Si-yan Zhan, Yong-hua Hu, Jie Jiang, Yan Zhang, Yong Huo, Da-fang Chen

**Affiliations:** Department of Epidemiology and Biostatistics, School of Public Health, Peking University Health Science Center, Beijing, 100191 China; Department of Cardiology, Peking University First Hospital, Beijing, 100034 China

## Abstract

**Background:**

Myocardial infarction (MI) is a serious complication of Coronary Artery Disease (CAD). Previous studies have identified genetic variants on chromosome 9p21 and 6p24 that are associated with CAD, but further studies need to be conducted to investigate whether these genetic variants are associated with the pathogenesis of MI. We therefore performed this study to assess the association between the risk of MI and SNP rs10757274 on chromosome 9p21 and SNP rs6903956 on chromosome 6p24, and to explore the gene-environment interactions in a Chinese population.

**Methods:**

A hospital-based case–control study, consisting of 502 MI patients and 308 controls, was conducted in a Chinese population. Demographic, behavioral information and clinical characteristics were collected, and genotyping of the two SNPs was performed using single base primer extension genotyping technology. The unconditional logistic regression (ULR) method was adopted to assess the association of the two SNPs with MI risk. Both generalized multifactor dimensionality reduction (GMDR) and ULR methods were applied to explore the effect of gene-environment interactions on the risk of MI.

**Results:**

After adjusting for covariates, it was observed that SNP rs10757274 on chromosome 9p21 was significantly associated with MI. Compared with subjects carrying the AA genotype, subjects carrying the GA or GG genotypes had a higher MI risk (OR_a_ = 1.52, 95% CI:1.06–2.19, *p*_*a*_ = 0.0227; OR_a_ = 2.40, 95% CI:1.51–3.81, *p*_*a*_ = 0.0002, respectively). Furthermore, a two-factor gene-environment interaction model of CDKN2A/B (rs10757274) and type 2 diabetes mellitus (T2DM) was identified to be the best model by GMDR (*p* = 0.0107), with a maximum prediction accuracy of 59.18%, and a maximum Cross-validation Consistency of 10/10. By using the ULR method, additive interaction analysis found that the combined effect resulted in T2DM-positive subjects with genotype GG/GA having an MI risk 4.38 times that of T2DM-negative subjects with genotype AA (OR_add_ = 4.38, 95% CI:2.56–7.47, *p*_add_ < 0.0001).

**Conclusions:**

These results show that gene polymorphism of CDKN2A/B (rs10757274) is associated with MI risk in a Chinese population. Furthermore, T2DM is likely to have an interaction with CDKN2A/B (rs10757274) that contributes to the risk of MI.

**Electronic supplementary material:**

The online version of this article (doi:10.1186/1471-2261-14-170) contains supplementary material, which is available to authorized users.

## Background

It has been reported that the global endemic of Coronary Artery Disease (CAD) is the leading cause of morbidity and mortality worldwide, due to serious complications such as myocardial infarction (MI) [1, 2]. Most researchers agree that, similarly to other complex diseases, multiple environmental factors, genetic factors and gene-environment interactions play important roles in determining the onset and development of CAD/MI [3]. There are many well-recognized environmental risk factors such as smoking, physical activity and body mass index (BMI). Genetic factors have also been considered to be substantial risk contributors to the pathogenesis of CAD/MI [4, 5]. Although previous studies have shown that the heritability of CAD ranges from approximately 40 to 60%, the genetic mechanism underlying the inherited component of CAD/MI is still not fully characterized [6].

In the past decade, there have been many studies on linkage and candidate gene association, however, these have only identified a limited number of genetic variants involved in the pathogenesis of CAD/MI. Genome-wide association studies (GWAS) have been a new approach since the completion of the Human Genome Project and the International HapMap Project, and have provided improved resolution in identifying genetic factors in relation to complex diseases. To date, many genome-wide studies have revealed that several genomic regions are strongly associated with an increased risk of CAD/MI [7–14], and it has been generally accepted that the association between genetic variants on chromosome 9p21 and the risk of CAD is the most robustly replicated finding in different ethnic populations [15–18]. However, several other studies have shown inconsistent results when the association between these genetic variants and MI was examined [19, 20]. Recently, a Chinese GWAS reported that chromosome 6p24 contains a novel susceptibility locus associated with CAD [14]. Some previous single studies have also suggested that genetic variants associated with cardiovascular disease might interact with hypertension or glycemic control [21, 22]. Therefore, we chose to study SNP rs10757274 on chromosome 9p21 and SNP rs6903956 on chromosome 6p24, to identify the association between these two susceptible genes and the risk of MI, and to explore gene-environment interactions in a Chinese population.

## Methods

### Study population

This project was designed as a hospital-based case–control study. Between June 2012 and May 2013, a total of 810 consecutive unrelated adult patients at Peking University First Hospital (Beijing, China) were enrolled in the study. Of these subjects, 502 patients undergoing coronary angiography for suspected or known coronary atherosclerosis were diagnosed with MI according to the universal definition of myocardial infarction [23], while 308 subjects were considered as the control group, who underwent coronary angiography for other reasons (mainly valvular heart disease) and had completely normal coronary arteries. Quanto software was used to measure the statistical power of this study; according to the frequencies of the minor allele (listed on NCBI) and the genetic effects that have been reported for these two SNPs, the statistical power of this study based on the current sample size is more than 0.90. The study was approved by the Ethics Committee of the Peking University Health Science Center, and all the subjects provided written informed consent for participation.

### Information collection

Demographic, behavioral information and clinical characteristics were collected from physicians and hospital records, including age, gender, ethnicity, smoking and drinking habits and some medical history information. A smoking habit was defined as current active smokers or those with a smoking history of at least10 packs per year, while a drinking habit was defined as consuming more than 50 g of alcohol per week for at least 6 months. Weight and height were measured when the subjects were lightly clothed and barefoot, and the BMI was calculated as the weight divided by the square of the height (kg/m^2^)). Overnight fasting blood samples were obtained from all participants, and measurements of the plasma concentrations of glucose, total cholesterol (TC, mmol/L), low density lipoprotein cholesterol (LDL-C, mmol/L), high density lipoprotein cholesterol (HDL-C, mmol/L) and triglyceride (TG, mmol/L) were conducted in the clinical laboratory of the Peking University First Hospital according to the standard biochemical methods. Combined with laboratory findings, type two diabetes mellitus (T2DM) was diagnosed according to the WHO diagnostic criteria from 1999 [24]; hypertension was defined as systolic blood pressure ≥140 mmHg and/or diastolic blood pressure ≥90 mmHg based on the average of two separate measurements, or current antihypertensive treatment; hyperlipidemia was diagnosed as fasting total cholesterol ≥200 mg/dL or low-density lipoprotein cholesterol ≥130 mg/dL.

### DNA preparation

EDTA anti-coagulated blood samples were drawn from every participant, and genomic DNA was extracted from the participants’ peripheral blood leucocytes by using the protein precipitation method following standard procedures. Each DNA sample was quantified using a Nanophotometer (Implen, Germany) and was diluted to a final concentration of 50 ng/uL.

### SNP genotyping

Genotyping of two SNPs (rs6903956 and rs10757274) was performed using the single base primer extension genotyping technology with the GenomeLab SNPstream genotyping system (Beckman Coulter Inc, Fullerton, CA, USA) following the manufacturer’s protocol [25]. Both of the SNPs had a call rate of greater than 95%, 10% of the blind duplicate samples were genotyped repeatedly and the rate of consistency was 100%.

### Statistical analysis

The descriptive and association analyses were performed using the SAS 9.1 software (SAS Institute Inc., Cary, NC). The continuous variables were shown as Mean ± SD, a two-sample *t*-test was adopted to compare their means. The comparison of categorical variables and testing of the Hardy-Weinberg Equilibrium (HWE) at each polymorphic locus in the control group was conducted using Pearson’s *χ*^*2*^ test. Unconditional logistic regression (ULR) was performed to estimate the association (Odds ratios (ORs) and 95% confidence intervals (CIs)) between individual polymorphisms and MI, adjusting for potential confounders, including gender, age, ethnicity, BMI, smoking and drinking habits. High dimensional gene-environment interactions were examined using the generalized multifactor dimensionality reduction method (GMDR, version 0.7, obtained from http://www.medicine.virginia.edu/clinical/departments/psychiatry/sections/neurobiologicalstudies/genomics/gmdr-software-request) [26], adjusting for age, gender, ethnicity, BMI, smoking and drinking habits as covariates. GMDR is a nonparametric method reducing high-dimensional data into one dimension. In this study, one- to five-factor models were constructed and the model with the highest prediction accuracy was defined as the “best model”. If the model was considered to be significant using the sign statistical test (*p <* 0.05), then a 1000 times permutation test was performed to validate the results. One dimensional multiplicative interaction was detected by ULR, adjusting for the potential confounders mentioned above. One dimensional additive interaction was estimated using the “Biological interaction calculating Excel” provided by Andersson et al. [27]. For two dichotomized risk factors, let OR_01_/OR_10_ represent exposure of each risk factor alone, let OR_11_ represent exposure of both risk factors, and let OR_00_ represent absence of both risk factors, which was used as the reference category. Three statistical indicators were calculated: relative excess risk of interaction (RERI = OR_11_ - (OR_01_ + OR_10_ -1)), attributable proportion of interaction (API = RERI/OR_11_), and the synergy index (S = (OR_11_ - 1)/[(OR_01_ - 1) + (OR_10_ - 1)]), along with their 95% CIs, to measure the additive interaction. If the 95% CIs of RERI and API did not include 0 and the 95% CI of the S index did not include 1, it can be considered that an additive interaction exists.

## Results

### Demographic, behavioral information and clinical characteristics of participants

A total of 810 unrelated Chinese subjects were enrolled in this study. The minimum and maximum ages among the participants were 28 and 88, respectively. The average age of the 502 cases was (63.66 ± 11.57) years, and the average age of the 308 controls was (61.87 ± 10.39) years. 64.92% of participants were male and 35.08% were female. The distributions of demographic, behavioral information and clinical characteristics of the subjects are listed in Table 1. There were no significant differences between the MI group and the control group in terms of mean BMI and distributions of ethnicity, hypertension and hyperlipidemia, while the mean age and frequencies of male gender, T2DM-positive, and smoking and drinking habits-positive were significantly higher in MI patients than in controls.

**Table 1:** **Demographic, behavioral information and clinical characteristics of participants**

Variable	Variable frequencies (%)			
	Controls (N = 308)	MI patients (N = 502)	t***/χ2***	***p***-value
**Age**	61.87 ± 10.39	63.66 ± 11.57	2.2823	0.0228
**BMI (kg/m** ^**2**^ **)**	25.84 ± 3.53	25.73 ± 3.09	0.4673	0.6404
**Gender**				
Male	47.73	77.09	73.2707	<.0001
Female	52.27	22.91		
**Ethnicity**				
Chinese Han	94.48	96.81	2.6567	0.1031
Ethnicity minority	5.52	3.19		
**Smoking habit**				
No	63.96	36.65	57.1381	<.0001
Yes	36.04	63.35		
**Drinking habit**				
No	74.68	61.75	14.3438	0.0002
Yes	25.32	38.25		
**T2DM**				
No	75.97	61.35	18.4275	<.0001
Yes	24.03	38.65		
**Hypertension**				
No	35.71	32.07	1.1376	0.2862
Yes	64.29	67.93		
**Hyperlipidemia**				
No	60.06	61.55	0.1778	0.6732
Yes	39.94	38.45		

### Association analysis of genetic polymorphisms with MI

Two SNPs, ADTRP (rs6903956) and CDKN2A/B (rs10757274) were studied in our association analysis. Both of their genotype distributions in the control group conformed to the HWE (*χ*^2^ = 2.756, *p* = 0.097; *χ*^2^ = 0.968, *p* = 0.325; respectively), which indicated these participants were from a homogeneous group. The genotype distributions of the two SNPs between MI patients and controls and their association with MI risk are available in Table 2, while the univariate associations between each of the two SNPs and the clinical characteristics listed in Table 1 for cases and controls are included in the Additional file 1: Table S1 and S2. Taking the subjects carrying the GG genotype for ADTRP (rs6903956) as a reference, the subjects carrying genotype AA/GA showed an increased risk of MI (OR = 1.51, 95% CI:1.01–2.23, *p* = 0.0423), but after adjusting for confounding factors including age, gender, ethnicity, BMI, smoking and drinking habits, the association between ADTRP (rs6903956) and MI risk was insignificant (OR = 1.49, 95% CI:0.97–2.29, *p* = 0.0678). According to the genetic effect and minor allele frequency of rs6903956 observed in this study, the statistical power based on the current sample size is 0.73. For CDKN2A/B (rs10757274), both before and after adjusting for confounding factors mentioned above, the subjects carrying GA or GG genotypes were all associated with MI risk (OR_a_ = 1.52, 95% CI:1.06–2.19, *p*_*a*_ = 0.0227; OR_a_ = 2.40, 95% CI:1.51–3.81, *p*_*a*_ = 0.0002; respectively), compared with subjects with the AA genotype. For rs10757274, the statistical power based on the current sample size is 0.97.

**Table 2:** **Association analysis of genetic polymorphisms with MI risk**

Chromosome location	Closest gene	SNPs	Genotypes	Genotype frequencies (%)		Without adjustment		With adjustment	
				Controls (N = 308)	MI patients (N = 502)	OR (95% CI)	***p***	OR _a_ (95% CI)	***p***_a_
6P24	ADTRP	rs6903956	GG	85.23	79.30	reference			
			GA	13.40	18.74	1.50 (1.00,2.27)	0.0525	1.55 (0.99,2.43)	0.0556
			AA	1.37	1.96	1.53 (0.47,5.03)	0.4813	1.04 (0.30,3.59)	0.9522
			AA/GA	14.78	20.70	1.51 (1.01,2.23)	0.0423	1.49(0.97,2.29)	0.0678
9P21	CDKN2A/B	rs10757274	AA	32.78	23.54	reference			
			GA	51.32	52.52	1.43 (1.02,1.99)	0.0374	1.52(1.06,2.19)	0.0227
			GG	15.89	23.94	2.10 (1.37,3.22)	0.0007	2.40 (1.51,3.81)	0.0002
			GG/GA	67.22	76.46	1.58 (1.15,2.18)	0.0045	1.72 (1.22,2.43)	0.0020

a: Adjusting for age, gender, ethnicity, BMI, smoking and drinking habits as covariates.

### Gene-environment interactions analysis

First, GMDR was used to explore the gene-environment interactions. ADTRP (rs6903956), CDKN2A/B (rs10757274) and clinical characteristics (T2DM, hypertension and hyperlipidemia) were included in the gene-environment interactions analysis, adjusting for age, gender, ethnicity, BMI, and smoking and drinking habits as covariates. The results of the GMDR analysis for one-factor to five-factor models are shown in Table 3. Among these results, the two-factor interaction model of CDKN2A/B (rs10757274) and T2DM was identified to be the best model, with a maximum prediction accuracy of 59.18%, and a maximum Cross-validation Consistency (CVC) of 10/10. This model was identified as significant using the sign test (*p* = 0.0107) and validated by a 1000 times permutation test (*p* < 0.001). These results indicate that a potential gene-environment interaction is likely to exist between T2DM and CDKN2A/B (rs10757274) affecting MI risk in this study.

**Table 3:** **GMDR models of gene-environmental interactions on MI risk**

Models ^a^	Prediction accuracy	CVC	***p***_sign_	***p***_perm_
T2DM	0.5815	10/10	0.0010	<0.001
T2DM, rs10757274	0.5918	10/10	0.0107	<0.001
T2DM, rs10757274, rs6903956	0.5796	10/10	0.0107	<0.001
T2DM, hyperlipidemia, rs10757274, rs6903956	0.5815	9/10	0.0547	<0.001
T2DM, hyperlipidemia, hypertension, rs10757274, rs6903956	0.5692	10/10	0.0547	0.008

^a^Adjusting for age, gender, ethnicity, BMI, smoking and drinking habits as covariates.

*p*_sign_: *p*-value from sign test.

*p*_perm_: *p*-value from 1000 times permutation test.

Second, to further reveal the potential gene-environment interaction mentioned above, ULR analysis was conducted to explore the possible multiplicative or additive gene-environment interactions between T2DM and CDKN2A/B (rs10757274) affecting MI risk. As shown in Table 4, there were no gene-environment multiplicative interactions between T2DM and CDKN2A/B (rs10757274) affecting MI risk (OR_multi_ = 1.63, 95% CI:0.78–3.44, *p*_*multi*_ = 0.197). After adjusting for age, gender, ethnicity, BMI, smoking and drinking habits as covariates, T2DM-negative subjects with the genotype GG/GA had a MI risk of 1.60 times that of T2DM-negative subjects carrying the genotype AA (OR_add_ = 1.60, 95% CI:1.04–2.46), and it was statistically significant (*p*_add_ = 0.0329); T2DM-positive subjects with genotype AA had a MI risk of 1.68 times that of T2DM-negative subjects carrying genotype AA (OR_add_ = 1.68, 95% CI:0.92 - 3.08), but it was not statistically significant (*p*_add_ = 0.0943). The combined effect resulted in T2DM-positive subjects with genotype GG/GA having an MI risk of 4.38 times that of T2DM-negative subjects carrying genotype AA (OR_add_ = 4.38, 95% CI:2.56 - 7.47), and this was statistically significant (*p*_add_ < 0.0001). Because the 95% CIs of RERI and API did not include 0, and the 95% CI of S did not include 1 (RERI = 2.10, 95% CI:0.18 - 4.03; API = 0.48, 95% CI:0.19 - 0.77; and S = 2.65, 95% CI:1.01 - 6.94, respectively), it can be considered that an additive gene-environment interaction exists between T2DM and CDKN2A/B (rs10757274) affecting the risk of MI.

**Table 4:** **Multiplicative and additive gene-environmental interactions analysis between T2DM and CDKN2A/B (rs10757274) on MI risk**

		Frequencies (%)					
Genotype	T2DM	Controls (N = 308)	MI patients (N = 502)	OR _add_^a^ (95% CI)	***p***_add_	OR _multi_^a^ (95% CI)	***p***_multi_
rs10757274						1.63(0.78,3.44)	0.197
AA	No	22.52	12.67	reference			
GG/GA		53.31	48.69	1.60 (1.04,2.46)	0.0329		
AA	Yes	10.26	10.87	1.68 (0.92,3.08)	0.0943		
GG/GA		13.91	27.77	4.38 (2.56,7.47)	<0.0001		
RERI				2.10 (0.18,4.03)			
API				0.48 (0.19,0.77)			
S				2.65(1.01,6.94)			

^a^Adjusting for age, gender, ethnicity, BMI, smoking and drinking habits as covariates.

*p*_*add*_: *p*-value from additive gene-environmental interactions analysis.

*p*_*multi*_: *p*-value from multiplicative gene-environmental interactions analysis.

## Discussion

Due to the fact that MI is the main complication of CAD and both of these diseases share common risk factors, both genetic variants and environmental risk factors associated with CAD are expected candidates affecting the risk of MI. In this case–control study, we chose two previously reported SNPs representing the genetic variants on 9p21 and 6p24 to investigate the association with MI. Our results indicate that a genetic variant of CDKN2A/B (rs10757274) on 9p21 is associated with MI after adjusting for conventional confounding factors, which consistently supports former results [10, 28, 29]. Further gene-environment interactions for ADTRP (rs6903956), CDKN2A/B (rs10757274), hypertension, dyslipidemia and T2DM were explored by GMDR and ULR methods, and the results from GMDR indicate that a gene-environment interaction is likely to exist between CDKN2A/B (rs10757274) and T2DM affecting the risk of MI. Further analytic results from ULR consistently support the existence of potential additive gene-environment interactions between CDKN2A/B (rs10757274) and T2DM, with a combined effect of increasing the risk of MI for T2DM-positive subjects with genotype GG/GA by 4.38 times that of T2DM-negative subjects carrying genotype AA. It had been previously reported that diabetes is a positive factor contributing to CAD/MI [30, 31]. T2DM, accounting for the majority of forms of diabetes, has also been considered a common disorder and an important risk factor for cardiovascular disease [32]. Recently, a report in a Chinese population also found that another genomic variant on 9p21 is associated with a risk of T2DM [33]. This is thought to be due to the CDKN2A and CDKN2B genes in this genomic region inhibiting CDK4 and CDK6, which has been demonstrated to influence β cell proliferation and mass, eventually leading to diabetes [34–36]. Meanwhile, a study published in 2008 reported an interaction between poor glycemic control and a 9p21 variant that increased the risk of CAD in T2DM [21]. Therefore, it is reasonable to consider that CDKN2A/B (rs10757274) not only increases the risk of MI, but also enhances the effects of T2DM on the risk of MI in this study.

In contrast to CDKN2A/B (rs10757274), we found that the genetic variant of ADTRP (rs6903956) on 6p24 was not associated with MI after adjusting for conventional confounding factors, which is inconsistent with recent research results reported in a Japanese population [37]. To our knowledge, only this solo Japanese research report has investigated the association between ADTRP (rs6903956) and MI to date, and the risk allele identified in this Japanese study differs from our study and two other studies conducted in Han Chinese and Japanese populations [14, 38]. Most former studies have focused on the identification of associations between ADTRP and CAD, but the results are controversial even in the same racial population [14, 39, 40]. It has been indicated that the underlying mechanism is due to regulation of the mRNA expression of the tissue factor pathway inhibitor (TFPI) by the ADTRP gene. Decreased TFPI expression would result in an increased rate of thrombosis and thus increase the risk of CAD [41]. Therefore, further studies are needed to completely reveal whether genetic variants on 6p24 are associated with the risk of MI.

There are inevitably some limitations to this study. First, as our study is based on a case–control design, it was difficult to avoid recall bias completely, and the results should be confirmed in prospective cohort studies. Second, our study investigated only a limited number of genetic variants related to MI, whereas many other genes and SNPs have been reported to contribute to MI susceptibility. Therefore, this study is not sufficient to evaluate genetic susceptibility to MI, and it would be useful for further studies to investigate more genetic polymorphisms associated with MI.

## Conclusion

In conclusion, the results of this study suggest that a gene polymorphism of CDKN2A/B (rs10757274) is associated with MI risk in a Chinese population. Furthermore, T2DM is likely to interact with CDKN2A/B (rs10757274) and contribute to the risk of MI.

## Electronic supplementary material

Additional file 1: Table S1: Univariate associations between rs6903956 and the characteristics listed in Table 1 for cases and controls. **Table S2.** Univariate associations between rs10757274 and the characteristics listed in Table 1 for cases and controls. (DOC 82 KB)

## Abbreviations

ULR: Unconditional logistic regression
GMDR: Generalized multifactor dimensionality reduction
CAD: Coronary Artery Disease
MI: Myocardial infarction
T2DM: Type two diabetes mellitus
BMI: Body mass index
GWAS: Genome-wide association studies
HWE: Hardy-Weinberg Equilibrium
ORs: Odds ratios
CIs: Confidence intervals.
