# Supplementary material for: Interaction of type 2 diabetes mellitus with Chromosome 9p21 rs10757274 polymorphism on the risk of myocardial infarction: a case–control study in Chinese population
Source: BMC Cardiovasc Disord. 2014 Nov 27;14:170. doi: 10.1186/1471-2261-14-170 (PMC4255939; doi:10.1186/1471-2261-14-170)
Supplement: Supplementary file 1 — Additional file 1: Table S1: Univariate associations between rs6903956 and the characteristics listed in Table 1 for cases and controls. Table S2. Univariate associations between rs10757274 and the characteristics listed in Table 1 for cases and controls. (DOC 82 KB) [file 12872_2014_813_MOESM1_ESM.doc]

**Additional file 1.**

| **rs6903956** | **Variable Frequencies (%)** | | | | | | | |
| --- | --- | --- | --- | --- | --- | --- | --- | --- |
|  | **controls(N=308)** | | | | **cases(N=502)** | | | |
|  | **GG** | **GA/AA** | **t/χ2** | ***p*** | **GG** | **GA/AA** | **t/χ2** | ***p*** |
| **age** | 62.06±10.49 | 60.53±10.62 | 0.88 | 0.3791 | 63.35±11.50 | 64.49±11.65 | 0.86 | 0.3888 |
| **BMI** | 25.90±3.59 | 26.08±2.81 | 0.36 | 0.7236 | 25.81±3.05 | 25.46±3.17 | 1.00 | 0.3181 |
| **gender** |  |  |  |  |  |  |  |  |
| Male | 45.97 | 53.49 | 0.83 | 0.3617 | 77.47 | 76.84 | 0.02 | 0.8960 |
| Female | 54.03 | 46.51 |  |  | 22.53 | 23.16 |  |  |
| **Ethnicity** |  |  |  |  |  |  |  |  |
| Chinese Han | 93.95 | 97.67 | 0.98 | 0.3228 | 96.98 | 97.89 | 0.23 | 0.6315 |
| Ethnicity minority | 6.05 | 2.33 |  |  | 3.02 | 2.11 |  |  |
| **Drinking habit** |  |  |  |  |  |  |  |  |
| No | 76.21 | 65.12 | 2.38 | 0.1230 | 62.91 | 52.63 | 3.34 | 0.0676 |
| Yes | 23.79 | 34.88 |  |  | 37.09 | 47.37 |  |  |
| **Smoking habit** |  |  |  |  |  |  |  |  |
| No | 64.52 | 62.79 | 0.05 | 0.8275 | 35.99 | 35.79 | 0.001 | 0.9712 |
| Yes | 35.48 | 37.21 |  |  | 64.01 | 64.21 |  |  |
| **T2DM** |  |  |  |  |  |  |  |  |
| No | 77.42 | 62.79 | 4.21 | 0.0401 | 64.01 | 50.53 | 5.77 | 0.0163 |
| Yes | 22.58 | 37.21 |  |  | 35.99 | 49.47 |  |  |
| **Hyperlipidemia** |  |  |  |  |  |  |  |  |
| No | 58.87 | 72.09 | 2.69 | 0.1011 | 59.62 | 66.32 | 1.42 | 0.2331 |
| Yes | 41.13 | 27.91 |  |  | 40.38 | 33.68 |  |  |
| **Hypertension** |  |  |  |  |  |  |  |  |
| No | 37.50 | 30.23 | 0.84 | 0.3606 | 32.14 | 32.63 | 0.01 | 0.9277 |
| Yes | 62.50 | 69.77 |  |  | 67.86 | 67.37 |  |  |

**Table S1: Univariate associations between rs6903956 and the characteristics listed in Table 1 for cases and controls**

**Table S2: Univariate associations between rs10757274 and the characteristics listed in Table 1 for cases and controls**

| **rs10757274** | **Variable Frequencies %** | | | | | | | |
| --- | --- | --- | --- | --- | --- | --- | --- | --- |
|  | **controls (N=308)** | | | | **cases (N=502)** | | | |
|  | **AA** | **GA/GG** | **t/χ2** | ***p*** | **AA** | **GA/GG** | **t/χ2** | ***p*** |
| **age** | 62.49±10.24 | 61.68±10.57 | 0.63 | 0.5280 | 62.36±11.62 | 64.12±11.50 | 1.45 | 0.1490 |
| **BMI** | 25.66±3.49 | 25.95±3.58 | 0.67 | 0.5059 | 25.66±2.79 | 25.75±3.19 | 0.29 | 0.7721 |
| **gender** |  |  |  |  |  |  |  |  |
| Male | 49.49 | 47.29 | 0.13 | 0.7189 | 76.07 | 77.63 | 0.12 | 0.7243 |
| Female | 50.51 | 52.71 |  |  | 23.93 | 22.37 |  |  |
| **Ethnicity** |  |  |  |  |  |  |  |  |
| Chinese Han | 96.97 | 93.10 | 1.87 | 0.1712 | 98.29 | 96.32 | 1.12 | 0.2900 |
| Ethnicity minority | 3.03 | 6.90 |  |  | 1.71 | 3.68 |  |  |
| **Drinking habit** |  |  |  |  |  |  |  |  |
| No | 69.70 | 76.35 | 1.54 | 0.2147 | 64.96 | 60.79 | 0.66 | 0.4173 |
| Yes | 30.30 | 23.65 |  |  | 35.04 | 39.21 |  |  |
| **Smoking habit** |  |  |  |  |  |  |  |  |
| No | 57.58 | 66.50 | 2.29 | 0.1302 | 29.91 | 38.95 | 3.14 | 0.0765 |
| Yes | 42.42 | 33.50 |  |  | 70.09 | 61.05 |  |  |
| **T2DM** |  |  |  |  |  |  |  |  |
| No | 68.69 | 79.31 | 4.10 | 0.0429 | 53.85 | 63.68 | 3.65 | 0.0560 |
| Yes | 31.31 | 20.69 |  |  | 46.15 | 36.32 |  |  |
| **Hyperlipidemia** |  |  |  |  |  |  |  |  |
| No | 58.59 | 61.58 | 0.25 | 0.6176 | 56.41 | 63.42 | 1.86 | 0.1724 |
| Yes | 41.41 | 38.42 |  |  | 43.59 | 36.58 |  |  |
| **Hypertension** |  |  |  |  |  |  |  |  |
| No | 39.39 | 33.00 | 1.19 | 0.2748 | 31.62 | 31.84 | 0.002 | 0.9647 |
| Yes | 60.61 | 67.00 |  |  | 68.38 | 68.16 |  |  |
